# Structure and Performance of Benzoxazine Composites for Space Radiation Shielding

**DOI:** 10.3390/molecules25184346

**Published:** 2020-09-22

**Authors:** Scott Winroth, Chris Scott, Hatsuo Ishida

**Affiliations:** 1 Material Answers LLC, 66 Buckskin Drive, Weston, MA 02493, USA; swinroth@materialanswers.com; 2 Department of Macromolecular Science and Engineering, Case Western Reserve University, 10900 Euclid Avenue, Cleveland, OH 44106, USA; hxi3@case.edu

**Keywords:** benzoxazine, multifunctional composite, polyethylene, carbon fiber, radiation shield, structural, galactic cosmic radiation

## Abstract

Innovative multifunctional materials that combine structural functionality with other spacecraft subsystem functions have been identified as a key enabling technology for future deep space missions. In this work, we report the structure and performance of multifunctional polymer matrix composites developed for aerospace applications that require both structural functionality and space radiation shielding. Composites comprised of ultra-high molecular weight polyethylene (UHMWPE) fiber reinforcement and a hydrogen-rich polybenzoxazine matrix are prepared using a low-pressure vacuum bagging process. The polybenzoxazine matrix is derived from a novel benzoxazine resin that possesses a unique combination of attributes: high hydrogen concentration for shielding against galactic cosmic rays (GCR), low polymerization temperature to prevent damage to UHMWPE fibers during composite fabrication, long shelf-life, and low viscosity to improve flow during molding. Dynamic mechanical analysis (DMA) is used to study rheological and thermomechanical properties. Composite mechanical properties, obtained using several standardized tests, are reported. Improvement in composite stiffness, through the addition of carbon fiber skin layers, is investigated. Radiation shielding performance is evaluated using computer-based simulations. The composites demonstrate clear advantages over benchmark materials in terms of combined structural and radiation shielding performance.

## 1. Introduction

Development of innovative multifunctional materials that combine structural functionality with other spacecraft subsystem functions, such as radiation shielding, has been identified as a key enabling technology for future deep space missions [1]. Radiation protection is identified by NASA as one of the four primary challenges that stand in the way of human travel to deep space [1]. Deep space contains ionizing radiation that can adversely affect the health and safety of astronauts and damage electrical components aboard spacecraft. High energy and heavy element (HZE) radiation from galactic cosmic rays (GCR) and solar energetic particles (SEP) is particularly harmful to humans and is known to result in carcinogenesis, degenerative tissue effects, acute radiation syndromes, and damage to the central nervous system [2].

To protect astronauts from harmful radiation, spacecraft incorporate passive shields that block, fragment, and deflect cosmic radiation. Conventional spacecraft designs typically utilize separate shield subsystems that are added onto, rather than incorporated into, the structure of the spacecraft [1]. The use of separate shielding materials adds parasitic weight and contributes to increased cost and complexity. Materials that are typically used to fabricate spacecraft structural components, such as aluminum, do not shield effectively against GCR and SEP radiation [3].

Radiation shields comprised of low atomic mass (low-*Z*) elements such as hydrogen are typically used to provide protection from GCR and SEP [4]. Low-*Z* elements place more nuclei in the path of incident radiation for a given shield areal density (mass per unit area) and are more effective at shielding against GCR and SEP than high atomic mass elements [5]. Polyethylene has been identified as the “best standard or non-novel” shielding material that provides useful radiation shielding performance and is practical in terms of cost, fabrication, and weight [5]. When molded or extruded using conventional techniques, polyethylene lacks adequate strength to be useful as a structural material. However, gel-spun ultra-high molecular weight polyethylene (UHMWPE) fibers provide excellent mechanical properties and can be used as reinforcement in polymer matrix composites. Use of UHMWPE fiber reinforcement is challenging due to its low melting temperature and low surface energy, which results in poor interfacial adhesion at the fiber-polymer interface [6]. Matrix resins are limited to those that can polymerize below the short duration temperature limit of the fiber (approximately 130 °C [7]).

In this paper, we report the structure and properties of polymer matrix composite materials, comprised of UHMWPE fiber reinforcement and a newly developed hydrogen-rich polybenzoxazine matrix, that are targeted for use in aerospace applications that require a combination of structural functionality and excellent space radiation shielding performance. Polybenzoxazines are organic thermosetting polymers belonging to the addition-curable phenolic resin family. Benzoxazines—the precursors to polybenzoxazines—are readily synthesized from a combination of three ingredients: a phenolic derivative, a primary amine, and formaldehyde. Flexible synthesis chemistry allows the chemical structure to be tailored at the molecular level to optimize material characteristics for targeted applications [8]. For example, polybenzoxazines have been formulated to provide low flammability [9], near-zero shrinkage [10], high thermal stability [11,12,13,14], and excellent mechanical properties [11,15,16,17].

**Figure 1:**
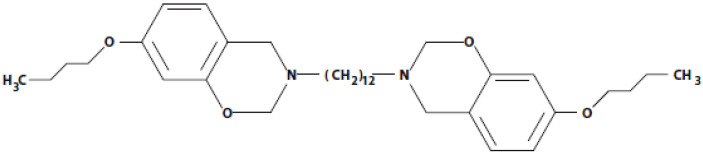
Molecular structure of 3BOP-daC12 benzoxazine monomer.

For the present application, benzoxazine resin chemistry was tailored to provide a superior combination of high hydrogen concentration, low polymerization temperature, long shelf-life, and low viscosity. A polymerization temperature below the short-duration temperature threshold of UHMWPE fiber (approximately 130 °C) was required to prevent physical degradation of the fiber’s mechanical properties, although the crystal melting temperature (144–152 °C) is slightly above this temperature [7]. This presented a significant challenge given that polybenzoxazines are typically polymerized in the temperature range of 160–220 °C [8]. A novel approach based on 3-butoxy phenol and a difunctional amine (1,12-diaminododecane) was developed to meet this challenge. The resulting benzoxazine monomer, abbreviated as 3BOP-daC12, is polymerizable at 120 °C and possesses high hydrogen concentration (estimated at 6.22 × 10^22^ H atoms/cm^3^). The molecular structure of the 3BOP-daC12 monomer is shown in Figure 1. Detailed information regarding the development and characterization of the benzoxazine monomer is reported elsewhere [18].

## 2. Results and Discussion

### 2.1. Polymerization of the Benzoxazine Resin and Preparation of Composites

Two types of composite samples—those comprised of UHMWPE fiber reinforcements and poly(3BOP-daC12) polybenzoxazine matrices (abbreviated henceforth as “UHMWPE/poly(3BOP-daC12)”) and those comprised of a three-layer sandwich structure consisting of two carbon fiber reinforced skin layers and a UHMWPE fiber reinforced core layer (abbreviated henceforth as “CF-UHMWPE-CF/poly(3BOP-daC12)”)—were prepared during this work. Poly(3BOP-daC12) was used as the matrix polymer for all sandwich composite layers. All composites were fabricated using a vacuum bagging process. This simple, low cost processing method was feasible due to the low viscosity of the resin. Work was conducted with a flat plate mold to provide a simple geometry amenable to process analysis as well as appropriate for specimen preparation for standardized mechanical tests. Plain-woven fabric of Spectra^®^ 1000 UHMWPE fibers (Honeywell International, Inc., Charlotte, NC, USA) was used as the reinforcement in all UHMWPE/poly(3BOP-daC12) composites and in the core layer of all sandwich composites. Plain-woven fabric of HexTow^®^ AS4 carbon fibers (Hexcel Corp., Stamford, CT, USA) was used in the skin layers of sandwich composites. A fiber volume fraction of 60% was targeted for all composites. Additional details regarding polymerization and composite processing are provided in Section 3.

### 2.2. Viscosity and Cure Kinetics of the Benzoxazine Resin

**Figure 2:**
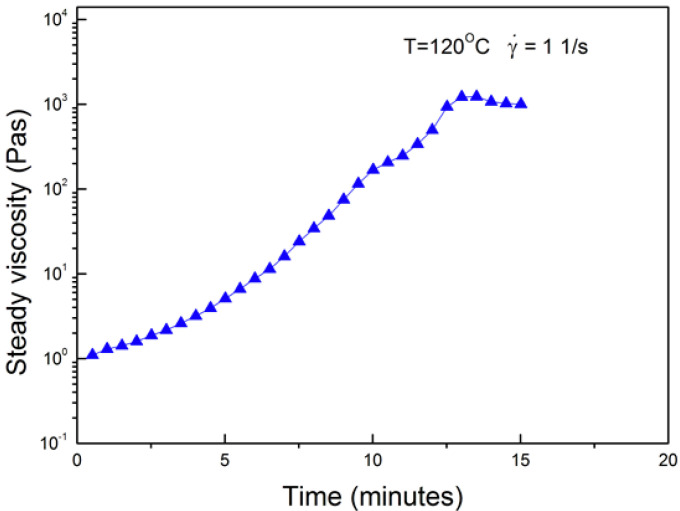
Isothermal shear viscosity for 3BOP-daC12 benzoxazine resin at a temperature of 120 °C.

Shear viscosity of the 3BOP-daC12 benzoxazine resin during cure was measured at an isothermal temperature of 120 °C using a steady shear viscometer. A plot of viscosity versus time is provided in Figure 2. A low initial shear viscosity value of approximately 1 Pa·s (1000 cP) was measured. Low resin viscosity is beneficial for many types of composite manufacturing processes including vacuum bagging, resin transfer molding (RTM), vacuum-assisted resin transfer molding (VARTM), and filament winding. For example, Campbell [19] identifies the preferred resin viscosity range for RTM processes as 0.10 to 0.50 Pa·s (100 to 500 cP) and a preferred viscosity of around 2.0 Pa·s (2000 cP) for filament winding processes.

**Figure 3:**
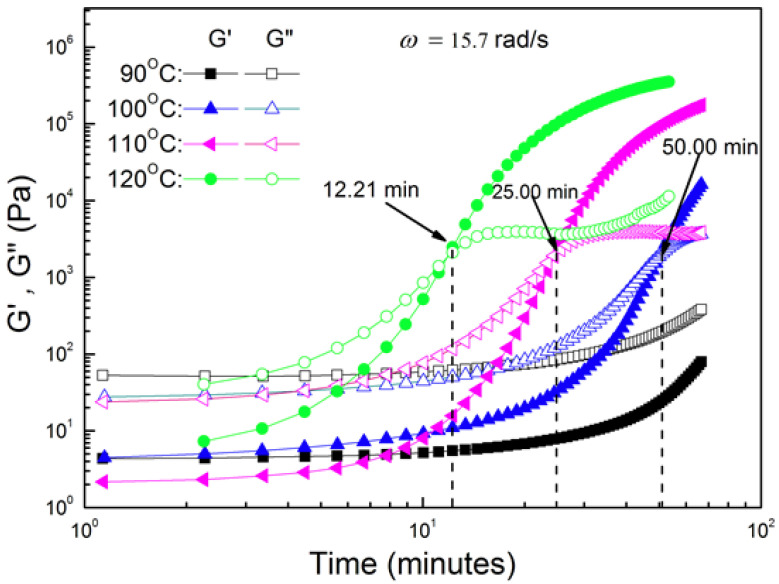
Dynamic mechanical analysis (DMA) spectra for isothermal cure of 3BOP-daC12 benzoxazine resin. The intersection of G’ and G” is defined as the gel point.

Resin gel time was measured using dynamic mechanical analysis (DMA). Gel time was determined according to ASTM D4473 (Standard Test Method for Plastics: Dynamic Mechanical Properties: Cure Behavior), which defines the gel point of a thermosetting resin or composite prepreg system as “the intersection of the elastic (G’) and viscous (G”) moduli”[20]. This G’/G” crossover criterion is not strictly the true gel time determination, as the time measured is dependent on the measurement frequency. A more rigorous determination is the Winter–Chambon criterion [21,22]. Nonetheless, the G’/G” crossover criteria is sufficient for the purpose of this project. Gel time was evaluated over a range of four isothermal polymerization temperatures: 90 °C, 100 °C, 110 °C, and 120 °C. Results from this testing are shown below graphically in Figure 3. A summary table of temperature versus gel time is provided in Table 1. The gel times of 12.2 min and 25.0 min measured at isothermal polymerization temperatures of 120 °C and 110 °C, respectively, are comparable to those of commercial aerospace epoxy resin systems. For example, the gel time of CYCOM^®^ 950-1, a low polymerization temperature (121 °C) aerospace-qualified epoxy resin, is specified as 13 min [23].

### 2.3. Resin Processing Window and Shelf-Life

**Figure 4:**
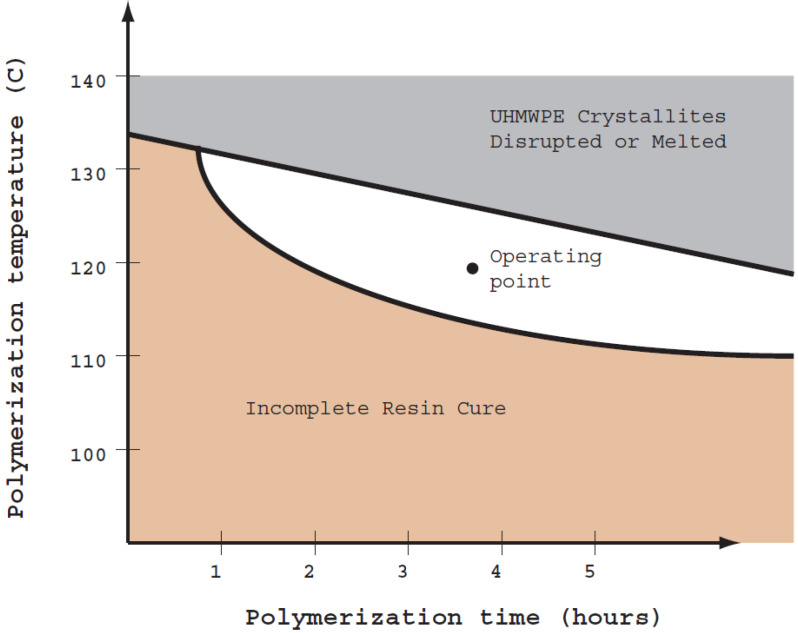
Graphical representation of the composite processing window for the vacuum bagging process.

Molding experiments, heat transfer modeling, and analytical testing provided the basis for establishing the processing window for the vacuum bagging process. The two most important variables for the processing window are the cycle time and the polymerization temperature. The processing window analysis is summarized in Figure 4, which provides the processing window in terms of these two variables. The operating point used for the current work is indicated in the center of the diagram and the estimated operating window is the white space around the operating point. The processing window is limited in the upward direction by the need to avoid melting or disruption of the UHMWPE crystallites, as indicated by the gray-colored region. The processing window is limited in the downward direction and to the left by the need to ensure polymerization of the resin, as indicated by the tan-colored region. The processing window as shown is open to the right, but in practice the longer polymerization cycle times in this direction would be avoided because they increase manufacturing costs.

The processing window for composite molding is narrowly confined in the cure temperature direction (y-axis in Figure 4). This is due to the key technical challenge associated with the polymerization characteristics of the resin: the two opposing requirements are that the resin must polymerize below the physical degradation temperature of UHMWPE and yet must have a sufficiently long shelf-life to be commercially viable.

The shelf-life of the 3BOP-daC12 monomer was evaluated over several months of storage and usage. A total of approximately 2.5 kg of 3BOP-daC12 monomer was prepared in eight batches and stored in a freezer until needed for sample fabrication. For composite fabrication, the monomer was removed from the freezer and remained at room temperature for 4 to 6 h during thawing, handling, and preparation of the composite. The monomer was then returned to the freezer. Samples were stored for longer than 3 months and experienced as many as 12 freeze/thaw cycles. The length of storage and repeated thawing and refreezing of the resin had no noticeable effects on viscosity, rheology, or processability. For comparison, a survey of fourteen commercial aerospace epoxy resins was conducted to establish an acceptable shelf-life benchmark. Manufacturer-specified shelf-life (under standard freezer storage conditions of −18 °C) was found to range from 6 months to 12 months. The 3BOP-daC12 monomer has an acceptable shelf-life for a commercial product.

### 2.4. Glass Transition Temperature as a Function of Post-Cure Heat Treatment

UHMWPE/poly(3BOP-daC12) glass transition temperature (T_g_) was measured by DMA as a function of post-cure heat treatment. Heat-treated specimens with heat treatment times ranging from 2 to 8 h were prepared and compared to a control specimen. Glass transition temperature results are reported in Table 2 using two definitions: 1) “DMA T_g_” according to ASTM D7028 [24] and 2) tan δ peak. The DMA T_g_ definition is frequently used to indicate the upper use temperature of composite materials and provides a more conservative value compared to the tan δ peak [24]. A trend of increasing T_g_ was observed as a result of heat treatment. Maximum T_g_ values of 84.2 °C (DMA T_g_) and 100.8 °C (tan δ peak) were obtained for the 8-h heat treatment specimen. Compared to the control specimen, the 8-h heat treatment specimen exhibited an increase in DMA T_g_ of 46.8 °C.

Storage modulus, E’, values at 30 °C are reported in Table 2 as well to provide an indication of UHMWPE fiber property degradation due to prolonged heat exposure. A trend of increasing E’ was observed for specimens heat-treated for 2 and 4 h. A slight reduction in storage modulus was observed after 6 h of heat treatment and a significant reduction in storage modulus was observed after 8 h. Reduction in E’ is likely an indication of UHMWPE fiber degradation. Given these results, the optimal heat treatment time appears to lie in the range of 4 to 6 h.

### 2.5. Mechanical Properties of Composites

Mechanical properties of UHMWPE/poly(3BOP-daC12) composites and CF-UHMWPE-CF/poly(3BOP-daC12) sandwich composites were evaluated using standardized tensile and flexural tests at room temperature. Additional mechanical property characterization—including compression, shear, and elevated and low temperature tensile testing—was conducted for UHMWPE/poly(3BOP-daC12) samples. Test method selection and conditions were guided by CMH-17 (Composite Materials Handbook) [25]. Detailed test descriptions are provided in Section 3.

UHMWPE/poly(3BOP-daC12) mechanical properties are summarized in Table 3. Maximum values for ultimate tensile strength (UTS) and tensile modulus were achieved at the cold temperature testing condition of −50 °C. The cold temperature UTS of 615 MPa is approximately 51% greater than the room temperature UTS of 493 MPa. This level of increase was somewhat surprising given that manufacturer literature for the UHMWPE fiber indicates only a 10% increase over room temperature tensile strength at −60 °C [7]. UTS of the UHMWPE/poly(3BOP-daC12) composite compares favorably to benchmark aerospace metals such as 6061-T6 aluminum alloy (UTS = 310 MPa) at room temperature. However, a significant drop in UTS and modulus was observed for the elevated temperature condition of 80 °C, which is above the glass transition temperature of poly(3BOP-daC12) when polymerized under standard conditions (i.e., no post-cure heat treatment). Open hole tensile strength was measured to provide a qualitative assessment of damage tolerance. The measured open hole tensile strength of 224 MPa represents a 55% reduction of the room temperature tensile strength (493 MPa), which is on par with reported reductions for carbon fiber/epoxy composites containing plain-woven reinforcements [26].

**Figure 5:**
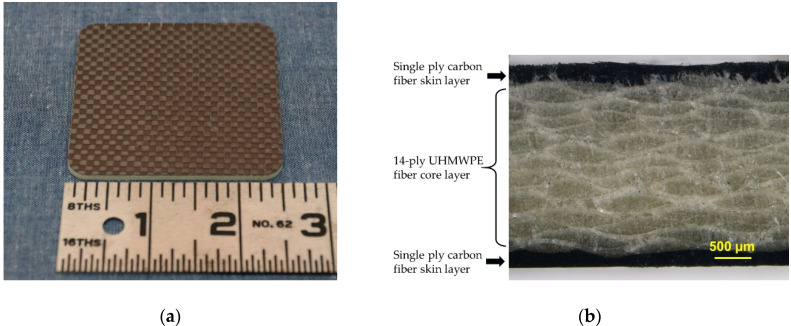
(**a**) Perspective view of a composite sample with carbon fiber skin layers; (**b**) cross-sectional micrograph of a composite with carbon fiber skin layers.

A composite structure incorporating skin layers reinforced with carbon fiber fabric was investigated as a means to achieve significant improvements in mechanical properties with minimal reduction in radiation shielding effectiveness. Carbon fiber is significantly stiffer (higher modulus) than UHMWPE fiber and was incorporated into the composite structure to provide increased tensile and flexural stiffness compared to UHMWPE/poly(3BOP-daC12) composites. For example, manufacturer’s data sheets indicate that HexTow^®^ AS4 carbon fiber has a tensile modulus of 231 GPa, whereas the tensile modulus of Spectra^®^ 1000 UHMWPE fiber is 103 GPa. Carbon fiber does not contain hydrogen, thus providing reduced GCR radiation shielding performance compared to UHMWPE fiber. It also has a higher mass density than UHMWPE fiber, which contributes to increased shield weight. For these reasons, use of carbon fiber in lightweight radiation shields should be minimized. CF-UHMWPE-CF/poly(3BOP-daC12) composite samples were fabricated with single layers of carbon fiber located in the outermost top and bottom skin layers to minimize carbon fiber content while maximizing improvement in mechanical properties. A perspective view of the composite and cross-section micrograph of the 3-layer design are shown in Figure 5.

Room temperature tensile and flexural properties of the UHMWPE/poly(3BOP-daC12) composite and the CF-UHMWPE-CF/poly(3BOP-daC12) sandwich composite are compared in Table 4. Dramatic increases in tensile modulus, flexural strength and flexural modulus were realized by adding carbon fiber skin layers. This represents a significant improvement in these properties with a minimal reduction in shielding efficacy. However, tensile strength was slightly reduced for the sandwich composite sample due to the unique failure mode described in more detail below.

**Figure 6:**
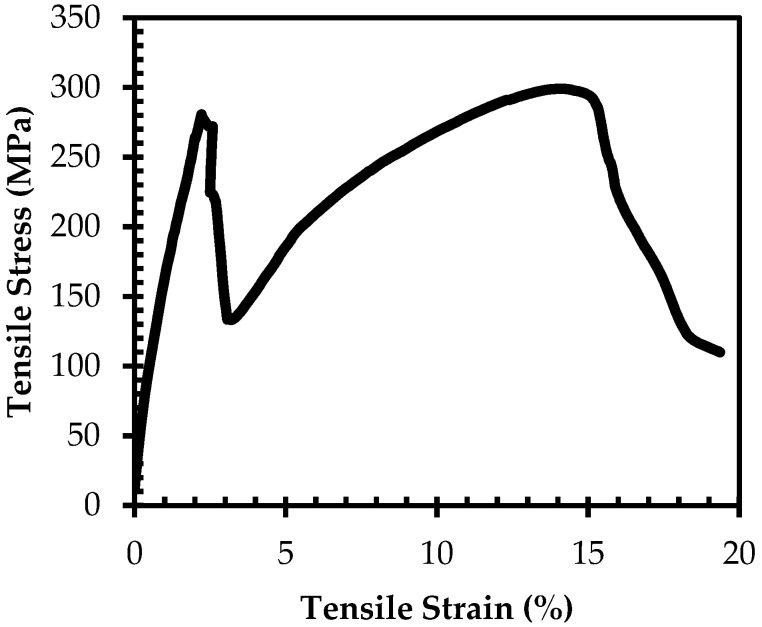
Example of dual-peak stress–strain curve obtained for CF-UHMWPE-CF/poly(3BOP-daC12) sandwich composite. Initial peak caused by failure of the carbon fiber skin layers followed by failure of the UHMWPE core layer.

During tensile testing, the carbon fiber reinforced skin layers in CF-UHMWPE-CF/poly(3BOP-daC12) sandwich composite specimens were observed to fail prior to the UHMWPE reinforced core layers resulting in stress-strain curves with dual peaks as shown in Figure 6. Skin layers failed within a tensile strain range of 1.2–2.2%, resulting in a sharp drop in tensile stress as the entire load was transferred to the core layer. Tensile stress then continued to increase, surpassing the initial peak, until failure of the core layer. UTS was recorded as the highest tensile stress over the entire stress-strain curve, which occurred at the second peak for all specimens. Core layers failed at tensile strain values between 13–16%; however, it should be noted that accurate strain measurements were only recorded for two out of six specimens due to dislodgement of the clip-on strain gage during initial failure of the skin layers. Compared to the tensile strain at failure for UHMWPE/poly(3BOP-daC12) samples, the strain at failure for CF-UHMWPE-CF/poly(3BOP-daC12) composite is around two times higher. Cyclic loading and unloading of UHMWPE fibers has been shown to increase maximum tensile strain, especially when the fibers are loaded at high stress values that approach the UTS of the fiber [27]. The initial loading of the UHMWPE fibers in the core layer followed by the subsequent unloading and reloading of the UHMWPE fibers after failure of the skin layers may have contributed to a shift in the maximum tensile strain and resulted in more ductile behavior.

### 2.6. Simulated Radiation Shielding Performance

NASA’s On-Line Tool for the Assessment of Radiation in Space (OLTARIS) [28] was utilized to estimate shielding performance for UHMWPE/poly(3BOP-daC12) and CF-UHMWPE-CF/poly(3BOP-daC12) composites. All composites were modeled having a fiber volume fraction of 60%. Materials were modeled as slabs with the exterior surface subjected to simulated GCR radiation exposure in a free space environment. The dose equivalent at the interior surface was then calculated and used to evaluate shielding performance, with lower dose values indicating better shielding performance. Further details about material modeling and simulation parameters are provided in Section 3.

For sandwich composites, the influence of skin layer thickness on shielding performance was investigated by modeling composites with different skin/core/skin volume ratios. Sandwich composites with six different skin/core/skin ratios were modeled and compared to a UHMWPE/poly(3BOP-daC12) composite having no skin layers. Additional materials—aluminum (6061 alloy, 2.7 g/cm^3^) and UHMWPE (TIVAR^®^ 1000 sheet, 0.93 g/cm^3^)—were also evaluated to provide performance benchmarks. All materials were evaluated at an areal density of 15 g/cm^2^.

Simulation results are summarized in Table 5. The naming convention used to identify sandwich composites in the table indicates the skin/core/skin volume ratio. A trend of increasing radiation dose (decreasing shielding performance) with increasing skin layer volume was observed for all sandwich composites. This trend is expected because the skin layers include carbon fiber rather than UHMWPE fiber, resulting in lower hydrogen concentration for the overall composite. When compared to the UHMWPE/poly(3BOP-daC12) composite, dose equivalent values for sandwich composites were 5.1–11.9% higher. However, all sandwich composites provided significantly better shielding performance (lower equivalent dose) than aluminum. The dose equivalent for the UHMWPE/poly(3BOP-daC12) composite was only 2.1% higher than that of UHMWPE, indicating only a slight reduction in shielding performance.

### 2.7. Evaluation of Multifunctional Performance

UHMWPE/poly(3BOP-daC12) composite materials were developed for use in aerospace applications requiring the combination of radiation protection and structural functionality. To evaluate the capabilities of UHMWPE/poly(3BOP-daC12) in this context, specific tensile strength and equivalent radiation dose were selected as performance metrics. Four additional materials—6061 aluminum alloy, CYCOM^®^ 934 epoxy, UHMWPE, and T300/CYCOM^®^ 934 composite—were evaluated to establish performance benchmarks for commercially available aerospace materials. CYCOM 934 epoxy (formerly Fiberite 934) was selected as a benchmark material based on its historical use in aerospace structures and radiation shielding research [29]. T300/CYCOM^®^ 934 is an aerospace composite material comprised of Toray T300 plain-woven carbon fiber fabric and CYCOM^®^ 934 epoxy. TIVAR^®^ 1000 was selected as a representative example of a commercial UHMWPE sheet material. Tensile strength and density values for benchmark materials were obtained from manufactures’ data sheets. Calculated specific strength values and properties used in specific strength calculations are listed in Table 6.

Equivalent radiation dose was estimated using NASA’s OLTARIS application. All materials were modeled as slabs with areal densities of 15 g/cm^2^ and were exposed to a simulated free space GCR environment. The GCR environment was modeled using the Badhwar-O’Neill 2010 Model and 1977 solar minimum event parameters, which were the same parameters used for simulations described earlier in this paper.

**Figure 7:**
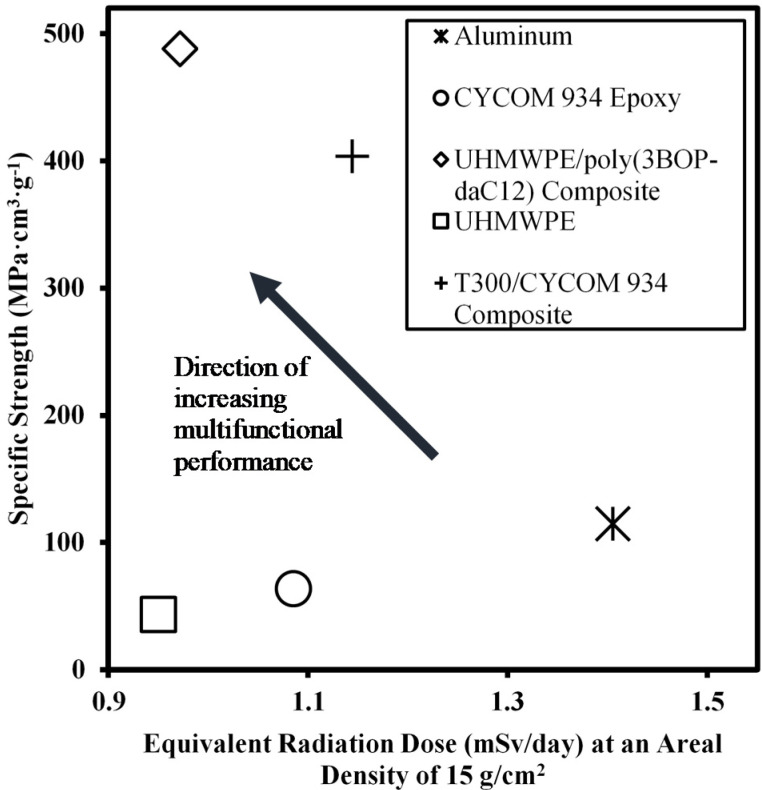
Plot of specific strength versus equivalent radiation dose for selected materials. Arrow indicates direction of increasing multifunctional performance in terms of higher specific strength and lower radiation dose.

Multifunctional capability was evaluated by plotting specific strength versus equivalent radiation dose. Note that a lower radiation dose indicates better shielding performance. As shown in Figure 7, the UHMWPE/poly(3BOP-daC12) composite provides a superior combination of high specific strength and excellent radiation protection that surpasses benchmark structural materials such as aluminum and T300/CYCOM^®^ 934 composite.

## 3. Materials and Methods

### 3.1. Materials

3-Butoxyphenol (97%) was purchased from 1717 CheMall Corp. (Mundelein, IL, USA) Paraformaldehyde (96% extra pure) was purchased from Sigma Aldrich Corp. (St Louis, MO, USA) 1,12-Diaminododecane (98%) was purchased from Tokyo Chemical Industry Company (Portland, OR, USA). Plain-woven Spectra^®^ 1000 UHMWPE fiber fabric (Style 932, 375 denier, 102 g/m^2^, 32 EPI × 31 PPI, scoured finish) was purchased from SAATI Americas Corp (Fountain Inn, SC, USA). Plain-woven HexTow^®^ AS4 carbon fiber fabric (3000 filament count per tow, 193 g/m^2^, 12.5 EPI × 12.5 PPI) was purchased from Composite Envisions LLC (Wausau, WI, USA).

### 3.2. Synthesis of 3BOP-daC12 Benzoxazine

3-Butoxyphenol, 1,12-diaminododecane, and para-formaldehyde were mixed in the stoichiometric amounts (mole ratio 2:1:4.2) in a round bottom flask. A slight excess of paraformaldehyde was used to offset losses from evaporation. Chloroform (5 mL per gram of reactants) was added to the round bottom flask. The solution was stirred at 60 °C for 7 h. The completed reaction product was washed three times with 1N NaOH and three times with distilled water. The product was dried over magnesium sulfate anhydrous overnight. The solution was filtered to remove the salt. After evaporating the solvent, the isomer mixture was dried in a vacuum oven to obtain a pale-yellow wax. The isomers contain butoxy groups at 5- and 7-positions with respect to the phenolic OH group and these isomers could not easily be separated in large quantity. NMR analysis shows that substitution at the 7-position is favored over substitution at the 5-position in a 3:1 ratio. The total reaction yield for the resulting benzoxazine isomeric monomer, abbreviated as 3BOP-daC12, was 75%.

### 3.3. Preparation of Composite Samples

**Figure 8:**
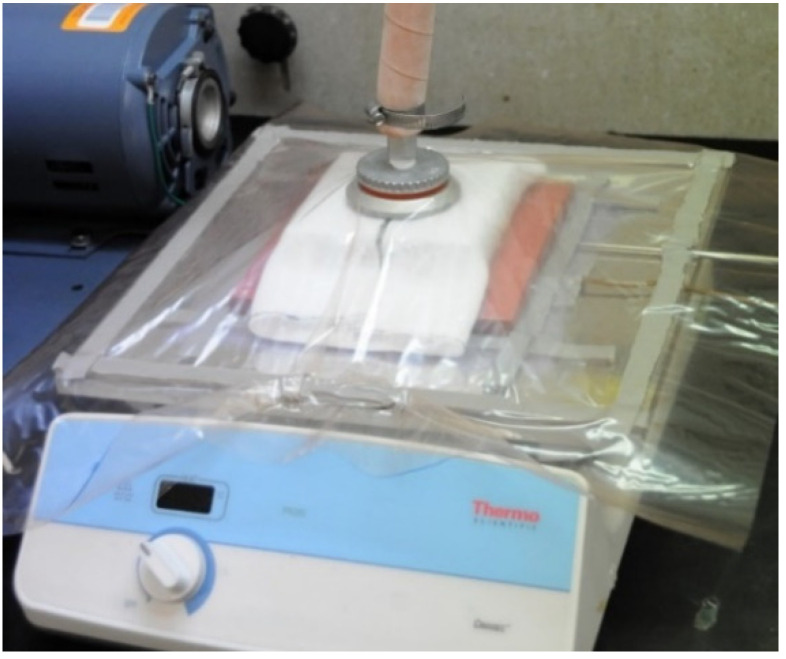
Photograph of the vacuum bagging mold assembly.

All composite samples were fabricated using a low-pressure vacuum bagging process. A flat mold, constructed from a 30.5 × 30.5 × 1.0 cm polished aluminum plate, was used as the primary fabrication tool. A hand lay-up process was used to apply materials to the working surface of the mold. Benzoxazine resin was applied to fabric plies using a spatula and plastic squeegee. A large ceramic hotplate was placed under the aluminum mold and used as the heat source. Consolidation and polymerization of all composites was achieved by simultaneously applying vacuum and heating the surface of the mold. The surface temperature of the aluminum mold was monitored and recorded throughout the polymerization cycle using surface-mounted thermocouples. A laboratory vacuum pump was used to apply vacuum (approximately 98 kPa or 29 in. Hg) to the mold during the polymerization and cooling cycles. The pump was connected using vacuum tubing and a through-bag vacuum connector located on the top surface of the breather layer. A photograph of a typical vacuum bag mold assembly is provided in Figure 8.

Polymerization conditions were selected so as to remain below the short duration temperature threshold of the UHMWPE fibers and prevent mechanical property degradation. An initial ramp-and-hold operation was performed at 80 °C to remove any excess moisture from the resin prior to polymerization. Mold temperature was increased from room temperature to 80 °C at a rate of about 2 °C/min and held at 80 °C for about one hour. Mold temperature was then increased to 120 °C at a rate of about 2 °C/min and held at 120 °C for at least two hours. The mold was removed from the heat source, placed on a cooling rack at room temperature, and allowed to cool to at least 60 °C prior to opening.

### 3.4. Radiation Shielding Simulations

Composite materials were modeled in OLTARIS by defining materials in terms of molecular mass percentage. Required inputs included molecular formula and mass percentage for the reinforcement and matrix as well as the overall composite density. Values used for each composite material are listed in Table 7. The density of poly(3BOP-daC12) was determined from measurements made on polymerized samples in accordance with ASTM D792. Fiber reinforcement densities were obtained from manufacturer’s data sheets. A reinforcement volume fraction of 60% was specified for each composite. Reinforcement and matrix mass percentages were calculated using density and volume percentage values. The theoretical fully-consolidated (void-free) density was calculated for each composite.

Materials were subjected to a simulated GCR free space environment at a distance of one astronomical unit (AU). Selection of GCR model parameters was guided by NASA’s Cross-Program Design Specification for Natural Environments (SLS-SPEC-159), which specifies that the Badhwar-O’Neill model and 1977 solar minimum event be used to model GCR environments when crew exposure to ionizing radiation is being evaluated [30].

Shield geometry was modeled in OLTARIS using the slab option, which utilizes straight ahead transport from one slab face to the other. CF-UHMWPE-CF/poly(3BOP-daC12) sandwich composite materials were simulated by constructing multilayer slabs having a first CF/poly(3BOP-daC12) skin layer, a core UHMWPE/poly(3BOP-daC12) layer, and a second CF/poly(3BOP-daC12) skin layer. Particles are transported from the simulated GCR free space environment through the material slab layers; generating fluxes/fluences at the material interface and the end boundary [28]. Dose equivalent radiation was calculated at the end boundary by OLTARIS using the International Commission on Radiological Protection (ICRP) 60 quality factor. All slabs were modeled and evaluated at areal densities of 15 g/cm^2^. For multilayer slabs used to model sandwich composites, the sum of areal densities for all layers was equal to 15 g/cm^2^.

### 3.5. Composite Characterization

#### 3.5.1. Tensile Test

Tensile testing was conducted on UHMWPE/poly(3BOP-daC12) composite samples to evaluate tensile strength, tensile modulus, Poisson’s ratio, and tensile strain at failure. A total of eighteen specimens were tested in accordance with ASTM D3039 using three different environmental conditions (6 specimens per condition). Testing was performed using an Instron 5985 universal test machine equipped with hydraulic wedge grips and an environmental test chamber. A strain gage was affixed to the center of each specimen using an adhesive. A cross-head speed of 2.0 mm/min was used for all specimens. Test specimens were laser cut from 254 × 254 mm composite plates that contained 8 plies of plain-woven UHMWPE fabric. Nominal test specimen dimensions were 254 × 25.4 × 1.5 mm.

Tensile testing was conducted on CF-UHMWPE-CF/poly(3BOP-daC12) sandwich composite samples to evaluate tensile strength and tensile modulus. A total of six specimens were tested in accordance with ASTM D3039 at room temperature. Testing was performed using an Instron 5566 universal test machine equipped with mechanical wedge grips. A cross-head speed of 2.0 mm/min was used for all specimens. Test specimens were machined from a 152.4 × 152.4 mm composite plate, which comprised a total of eight plies of reinforcement fabric (one ply of carbon fiber fabric in each skin layer and six plies of UHMWPE fabric in the core layer). Nominal test specimen dimensions were 152.4 × 12.7 × 1.5 mm. Note that smaller specimens were used for the sandwich composite compared to the UHMWPE/poly(3BOP-daC12) composite due to limited resin availability at the time of fabrication. Specimen dimensions still adhered to ASTM D3039 guidelines.

#### 3.5.2. Open-Hole Tensile Test

Open-hole tensile testing was conducted on UHMWPE/poly(3BOP-daC12) composite samples to evaluate open-hole tensile strength and assess damage tolerance. A total of six specimens were tested in accordance with ASTM D5766 at room temperature. Testing was performed using an Instron 5985 universal test machine equipped with hydraulic wedge grips. A cross-head speed of 2.0 mm/min was used for all specimens. Test specimens were laser cut from 254 × 254 mm composite plates that contained 16 plies of plain-woven UHMWPE fabric. Nominal test specimen dimensions were 254 × 38.1 × 2.9 mm with a 6.35 mm diameter center hole.

#### 3.5.3. Flexural Test

Flexural testing was performed on UHMWPE/poly(3BOP-daC12) and CF-UHMWPE-CF/poly(3BOP-daC12) composite samples to determine flexural strength and flexural modulus at room temperature. A total of ten UHMWPE/poly(3BOP-daC12) specimens and six CF-UHMWPE-CF/poly(3BOP-daC12) specimens were tested in accordance with Procedure A of ASTM D790 using a standard span-to-thickness ratio of 16:1. Testing was conducted using an Instron 5566 universal test machine equipped with a three-point bending fixture. UHMWPE/poly(3BOP-daC12) specimens were laser cut from a 152.4 × 152.4 mm composite plate that contained 16 plies of plain-woven UHMWPE fabric. CF-UHMWPE-CF/poly(3BOP-daC12) specimens were machined from a 152.4 × 152.4 mm composite plate comprised of sixteen fabric plies (one ply of carbon fiber fabric in each skin layer and fourteen plies of UHMWPE fabric in the core layer) using a waterjet cutter. Nominal dimensions for all test specimens were 127 × 12.7 × 3.0 mm.

#### 3.5.4. Compressive Test

Compression testing was conducted on UHMWPE/poly(3OBPH-dac12) composite samples to evaluate compression strength and modulus. A total of six specimens were tested in accordance with ASTM D6641 at room temperature. Testing was performed using an Instron 5985 universal test machine equipped with a Zwick hydraulic composite compression fixture. A cross-head speed of 1.3 mm/min was used for all specimens. Test specimens were machined from a 152.4 × 152.4 mm composite plate containing 32 plies of plain-woven UHMWPE fabric. Machining was accomplished using a waterjet cutter. Nominal test specimen dimensions were 139.7 × 12.7 × 5.6 mm.

#### 3.5.5. In-Plane Shear Test

In-plane shear testing was conducted on UHMWPE/poly(3BOP-daC12) composite samples to evaluate shear strength and shear modulus. A total of six specimens were tested in accordance with ASTM D3518 at room temperature. Testing was conducted using an Instron 5985 universal test machine equipped with hydraulic wedge grips. A cross-head speed of 2.0 mm/min was used for all specimens. Test specimens were laser cut from a 152.4 × 152.4 mm composite plate containing 8-plies of plain-woven UHMWPE fabric. Nominal test specimen dimensions were 254 × 25.4 × 1.5 mm.

#### 3.5.6. Short Beam Shear Test

Short beam shear testing was performed on UHMWPE/poly(3BOP-daC12) composite samples to evaluate short beam shear strength. A total of six specimens were tested in accordance with ASTM D2344 at room temperature. Testing was conducted using an Instron 5985 universal test machine equipped with a short beam shear test fixture. A cross-head speed of 1.0 mm/min was used for all specimens. Test specimens were machined from a 152.4 × 152.4 mm composite plate containing 32 plies of plain-woven UHMWPE fabric. Machining was accomplished using a waterjet cutter. Nominal test specimen dimensions were 38.1 × 12.7 × 5.6 mm.

#### 3.5.7. Dynamic Mechanical Analysis

Dynamic mechanical properties of UHMWPE/poly(3BOP-daC12) composites were measured using a DMA Q800 dynamic mechanical analyzer (TA Instruments, New Castle, DE, USA). Five composite specimens with nominal dimensions of 60 × 12 mm were cut from a 254 × 254 × 1.5 mm composite plate sample that had been polymerized using a standard two-hour polymerization cycle. Four of the specimens were further subjected to post-cure heat treatments ranging from 2 to 8 h.

Heat treating was carried out in a laboratory convection oven that was pre-heated to 120 °C. One specimen was removed from the oven every two hours; yielding specimens with heat-treatment times of 2, 4, 6, and 8 h. Each specimen was constrained between two flat aluminum plates to prevent warping during heat-treatment and cooling.

DMA specimens were tested in isostrain mode at a constant strain amplitude of 0.1% and a frequency of 1 Hz. Load was applied using a dual cantilever fixture. Temperature was ramped from room temperature to 140 °C at a heating rate of 5 °C/min.

## 4. Conclusions

Novel polymer matrix composites, including UHMWPE fiber reinforcement and hydrogen-rich polybenzoxazine, have been developed to meet the need for a multifunctional material that provides structural functionality and radiation protection. An evaluation of the composite’s multifunctional capability was performed and demonstrates a clear advantage over benchmark materials in terms of combined structural and radiation shielding performance. Compared to aluminum, the UHMWPE/poly(3BOP-daC12) composite provides a 325% increase in specific strength and an estimated 31% reduction in equivalent radiation dose. Composite samples containing carbon fiber skin layers further demonstrated a 144% increase in tensile modulus and a 227% increase in flexural modulus. OLTARIS radiation simulations also demonstrate that UHMWPE/poly(3BOP-daC12) shielding performance approaches that of pure polyethylene, with the difference estimated to be less than 3% at an areal density of 15 g/cm^3^.

Empirical data related to radiation shielding performance are anticipated from on-going testing in space. UHMWPE/poly(3BOP-daC12) composite samples are being tested on the International Space Station (ISS) as part of Materials International Space Station Experiment 12 (MISSE-12). MISSE-12 was installed on the MISSE-Flight Facility (FF) on the outside of the ISS in November 2019 and is scheduled to have one year of space exposure.

## 5. Patents

Scott, C. E.; Ishida, H.; Winroth, S. A. “Polybenzoxazine resins with high hydrogen content, and composites therefrom” U.S. Patent Appl. No. 16/375,415 published as US 2019/0309129A1, 10 October 2019.

## Figures and Tables

**Table 1:** Summary of resin gel time as a function of isothermal polymerization temperature.

Isothermal Polymerization Temperature	Gel Time
90 °C	>60 min
100 °C	50.0 min
110 °C	25.0 min
120 °C	12.2 min

**Table 2:** Summary of composite glass transition temperature and storage modulus as functions of post-cure heat treatment.

Post-Cure Heat Treatment Time	Glass Transition Temperature, DMA T_g_	Glass Transition Temperature, Tan δ Peak	Storage Modulus, E’ at 30 °C
0 h (control)	37.4 °C	55.1 °C	8.0 GPa
2 h	65.2 °C	83.6 °C	9.9 GPa
4 h	74.4 °C	92.2 °C	10.4 GPa
6 h	79.4 °C	96.9 °C	10.3 GPa
8 h	84.2 °C	100.8 °C	8.8 GPa

**Table 3:** Summary of ultra-high molecular weight polyethylene (UHMWPE)/poly(3BOP-daC12) composite mechanical properties.

Property	Test Method	Composite Layup ^1^	Test Condition ^2^		
			CTD	RTD	ETW
Tensile Strength, 0°	ASTM D3039	[0]_8_	615 MPa	493 MPa	239 MPa
Tensile Modulus (Chord, 0.1–0.3% Strain), 0°	ASTM D3039	[0]_8_	13.3 GPa	8.8 GPa	2.8 GPa
Tensile Strain at Failure, 0°	ASTM D3039	[0]_8_	-	6.9%	-
Poisson’s Ratio	ASTM D3039	[0]_8_	0.100	0.118	0.116
Open Hole Tensile Strength	ASTM D5766	[45/0/−45/90]_2S_	-	224 MPa	-
Compressive Strength, 0°	ASTM D6641	[0]_32_	-	27.5 MPa	-
Compressive Modulus, 0°	ASTM D6641	[0]_32_	-	19.4 GPa	-
Flexural Strength	ASTM D790	[0]_16_	-	63.1 MPa	-
Flexural Modulus	ASTM D790	[0]_16_	-	7.8 GPa	-
Short Beam Shear Strength	ASTM D2344	[0]_32_	-	5.5 MPa	-
In-Plane Shear Strength	ASTM D3518	[45/−45]_2S_	-	9.0 MPa	-
In-Plane Shear Modulus	ASTM D3518	[45/−45]_2S_	-	0.7 GPa	-

^1^ Orientation codes and stacking sequence notation defined by CMH-17 (Composite Materials Handbook) [25]. ^2^ CTD = cold temperature, dry (−50 °C); RTD = room temperature, dry (23 °C, 50% RH); ETW = elevated temperature, wet (80 °C, 85% RH).

**Table 4:** Comparison of room temperature tensile and flexural properties for UHMWPE/poly(3BOP-daC12) composites and CF-UHMWPE-CF/poly(3BOP-daC12) sandwich composites.

Property	Test Method	UHMWPE/poly(3BOP-daC12)	CF-UHMWPE-CF/poly(3BOP-daC12)	Percent Change
Tensile Strength, 0°	ASTM D3039	493 MPa	303 MPa	−39%
Tensile Modulus (Chord, 0.1–0.3% Strain), 0°	ASTM D3039	8.8 GPa	21.5 GPa	+144%
Flexural Strength	ASTM D790	63.1 MPa	120.4 MPa	+90%
Flexural Modulus	ASTM D790	7.8 GPa	25.5 GPa	+227%

**Table 5:** Comparison of On-Line Tool for the Assessment of Radiation in Space (OLTARIS) simulation results.

Material Name ^1^	Total Skin Layer Vol%	Dose Equivalent ^2^ (mSv/day)	Percent Change in Dose Equivalent Compared to UHMWPE/poly(3BOP-daC12) Composite
UHMWPE	N/A	0.951	−2.1%
UHMWPE/poly(3BOP-daC12) Composite	0%	0.971	-
2.5/95/2.5 Sandwich Composite	5%	1.021	+5.1%
5/90/5 Sandwich Composite	10%	1.032	+6.2%
10/80/10 Sandwich Composite	20%	1.049	+8.0%
15/70/15 Sandwich Composite	30%	1.063	+9.4%
20/60/20 Sandwich Composite	40%	1.075	+10.7%
25/50/25 Sandwich Composite	50%	1.087	+11.9%
Aluminum	N/A	1.405	+44.6%

^1^ Sandwich composite naming convention indicates the skin/core/skin volume ratio. ^2^ All materials evaluated at an areal density of 15 g/cm^2^.

**Table 6:** Calculated specific strength values and properties used in specific strength calculations.

Material	Density (g/cm^3^)	Tensile Strength (MPa)	Specific Strength (MPa·cm^3^·g^−1^)
Aluminum	2.70	310	115
CYCOM^®^ 934 Epoxy	1.30	83	64
T300/CYCOM^®^ 934 Composite	1.58	638	404
TIVAR^®^ 1000 UHMWPE	0.93	40	43
UHMWPE/ Poly(3BOP-daC12) Composite	1.01	493	488

**Table 7:** Properties used to define OLTARIS composite material models.

		UHMWPE/poly(3BOP-daC12) Composite	CF/poly(3BOP-daC12) Composite
Reinforcement	Type	UHMWPE fiber	Carbon fiber
	Molecular formula	CH_2_	C
	Density	0.97 g/cm^3^	1.79 g/cm^3^
	Volume %	60%	60%
	Mass %	57.62%	71.50%
Matrix	Type	poly(3BOP-daC12)	poly(3BOP-daC12)
	Molecular formula	C_36_H_56_N_2_O_4_	C_36_H_56_N_2_O_4_
	Density	1.07 g/cm^3^	1.07 g/cm^3^
	Volume %	40%	40%
	Mass %	42.38%	28.50%
Composite Density		1.01 g/cm^3^	1.50 g/cm^3^
